# Metabolic Derangements Contribute to Reduced sRAGE Isoforms in Subjects with Alzheimer's Disease

**DOI:** 10.1155/2018/2061376

**Published:** 2018-02-22

**Authors:** Kelly N. Z. Fuller, Edwin R. Miranda, John P. Thyfault, Jill K. Morris, Jacob M. Haus

**Affiliations:** ^1^Department of Kinesiology and Nutrition, University of Illinois at Chicago, Chicago, IL, USA; ^2^Department of Molecular & Integrative Physiology, University of Kansas Medical Center, Kansas City, KS, USA; ^3^Department of Neurology, University of Kansas Medical Center, Fairway, KS, USA

## Abstract

Although there is evidence for metabolic dysfunction and chronic inflammation in Alzheimer's disease (AD), circulating levels of soluble receptor for advanced glycation end products (sRAGE) and the receptor for advanced glycation end products (RAGE) ligand S100B have not been characterized. sRAGE is an important mediator in disease as it can act as a ligand decoy for RAGE and attenuate downstream inflammatory signaling. Cognitively healthy elderly and AD participants with and without type 2 diabetes (*n* = 135) were stratified according to the clinical dementia rating (CDR; 0 = normal cognition (NC); ≥0.5 = AD). Total serum sRAGE, endogenous secretory RAGE (esRAGE), and S100B were assayed via ELISAs, and cleaved RAGE (cRAGE) and the cRAGE : esRAGE ratio were calculated. cRAGE : esRAGE was lower in AD compared to NC (*p* < 0.05). Metabolic substratifications were used to investigate the factors that influence sRAGE pathology in AD. Stratification by BMI classification, median fat mass, median HOMA-IR, median insulin, and median amylin were all metabolic or anthropometric factors which significantly interacted with sRAGE profiles within AD subjects. There were no significant differences in serum S100B between groups. These characterizations of sRAGE contribute evidence to the link between impaired metabolism and cognitive decline due to AD.

## 1. Introduction

Research has identified impaired glucose metabolism and type 2 diabetes (T2DM) as risk factors for cognitive impairment (CI) and Alzheimer's disease (AD) [1–4]. Further, growing evidence of shared biochemical features between AD, T2DM, and insulin resistance (IR) suggests that AD may fundamentally be characterized as a metabolic disorder [5–7]. The recent increase in life expectancy has led to a greater population of individuals who are experiencing comorbid T2DM and AD, as prevalence of both T2DM and AD increases with age [8, 9]. Therefore, it is important to investigate the cellular and metabolic profiles of these individuals to give insight into the potential mechanisms underlying the onset of AD, which have yet to be fully elucidated. Recent evidence suggests that the receptor for advanced glycation end products (RAGE) may serve as an important mechanistic link [10].

RAGE is a transmembrane receptor of the immunoglobulin superfamily, thought to have evolved from a family of cell adhesion molecules [11]. RAGE has a diverse set of ligands, which include advanced glycation end products (AGEs), beta-amyloid (A*β*), and members of the S100/calgranulin protein family [12, 13]. These ligands and the RAGE protein are increased in the postmortem brains of AD patients, and interactions between RAGE and its ligands are related to the pathophysiology of chronic disease [14, 15]. More specifically, it has been documented that in the AD brain, there is an overexpression of glial-derived factors including the S100B protein [16]. At high concentrations, S100B accumulates at the surface of RAGE and activates the receptor [17]. Activation of RAGE initiates oxidative stress signaling pathways and inflammation through the transcription factor nuclear factor-kappa B (NF-*κ*B) [18]. Chronic inflammation and excessive ROS production are hallmarks of diabetes and metabolic disorders, and increased oxidative stress in the brain is a major inducer of AD [19–21]. RAGE is further implicated in the pathogenesis of AD as it plays a critical role in cerebral A*β* production and accumulation, neuronal degeneration, diminished synaptic transmission, and the formation of fibrous tangles [10]. These biological factors are considered to be the main contributors to the cognitive impairment characteristic of AD [22].

RAGE exists in two major forms: full-length, membrane bound RAGE and a circulating form termed soluble RAGE (sRAGE). Further, sRAGE is comprised of two distinct isoforms produced by unique mechanisms. Cleaved RAGE (cRAGE) is produced via proteolytic cleavage of the RAGE ectodomain by matrix metalloproteases and endogenous secretory RAGE (esRAGE) results from alternative splicing of RAGE pre-mRNA [23, 24]. Both isoforms of sRAGE lack the transmembrane and cytosolic domains necessary for signal transduction and act as a ligand decoy, thus attenuating intracellular RAGE signaling and subsequent inflammation [25]. Individuals with obesity, diabetes, or CI alone present with reduced circulating sRAGE profiles compared to lean healthy controls [26–29]. This evidence has led to the emergence of sRAGE as a suggested biological marker of both glucose tolerance status [29] and cognitive decline [30, 31].

Despite the well-established link between RAGE and the pathophysiology of T2DM and AD, the effects of concurrent metabolic and cognitive derangement on sRAGE remain unknown. The purpose of this study was to investigate plasma levels of sRAGE and the RAGE ligand S100B in individuals with metabolic disturbances such as obesity, impaired fasting glucose, elevated amylin, and T2DM comorbid with AD. Our hypothesis was that individuals with concurrent metabolic derangement and AD would display decreased levels of sRAGE isoforms and elevated levels of S100B. Further, we hypothesized that plasma sRAGE levels would be inversely related to that of S100B.

## 2. Methods

### 2.1. Participants

All participants (*n* = 135) included in this cross-sectional analysis were part of the University of Kansas Alzheimer's Disease Center Clinical Cohort and provided written informed consent according to the University of Kansas Medical Center's institutional guidelines. Eligible participants were postmenopausal, on stable medicine, and able to provide informed consent. Exclusion criteria included diagnosis of type 1 diabetes, clinically significant depression, or any neurodegenerative disorders other than AD. We a priori included samples from an approximately equal number of T2DM participants in both the ND and AD groups.

### 2.2. Clinical Characterization and Measurements

Individuals were clinically assessed, and dementia severity was characterized using the Clinical Dementia Rating (CDR). All subjects underwent neuropsychometric testing. Diagnosis was confirmed at a consensus diagnosis conference based on clinical information and neuropsychometric performance. Participants were considered to have normal cognition (NC) if they were CDR 0 with no clinically significant deficits on cognitive testing. All AD subjects were diagnosed as either mild cognitive impairment due to probable AD or dementia due to probable AD at the time of the blood draw. All AD subjects had a CDR of 0.5 or higher.

For characterization by apolipoprotein *ε*4 (APOE4) genotype, DNA was isolated from whole blood and sent to the National Cell Repository for Alzheimer's Disease (NCRAD) for genotyping. Participants were classified as either APOE4 carriers or noncarriers based on the presence of a single APOE4 allele.

American Diabetes Association (ADA) criteria were used to characterize the fasting glucose status (FGS) of participants. Participants were categorized as having normal fasting glucose (NFG, <100 mg/dL), impaired fasting glucose (IFG, 100–125 mg/dL), or diabetes (T2DM, >125 mg/dL). Further, homeostatic model assessment of insulin resistance (HOMA-IR) was computed as a measure of insulin sensitivity [32].

World Health Organization (WHO) criteria were used to characterize the body mass index (BMI) of participants. Participants were classified as lean (18.5–24.9 kg/m^2^), overweight (25–29.9 kg/m^2^), or obese (>30 kg/m^2^). Body composition was assessed via dual-energy X-ray absorptiometry (DEXA, Lunar Prodigy, version 11.2068) for measures of lean body mass and fat mass.

### 2.3. Serum Analysis

Participants reported to the clinic following an overnight fast, and blood was collected via venipuncture into serum vacutainer tubes containing clot activator. Blood was processed for serum, and glucose was measured using a YSI 2300 Stat Plus Glucose Lactate Analyzer. All remaining serum was stored at −80°C until further analysis. Insulin (Genway), amylin (Millipore), S100B (Millipore), sRAGE (R&D), and esRAGE (As One International) were quantified using ELISA per the manufacturer's protocol. Serum cRAGE was computed by subtracting esRAGE from the total plasma sRAGE pool as previously described [33], and the cRAGE : esRAGE ratio was subsequently calculated [29].

### 2.4. Statistics

All data were tested for normality using Shapiro-Wilk's test. Nonnormally distributed data was log-transformed and retested for normality. The Student's *t*-test was used to compare serum sRAGE and S100B profiles between the AD and NC groups. An analysis of variance (ANOVA) and Bonferroni post hoc analysis were used to determine significant differences in sRAGE isoforms between cognition groups and across important metabolic factors. An analysis of covariance (ANCOVA) was used following the ANOVA to determine the effects of age and sex, two well-established influences in AD pathology. Pearson's correlation coefficient was used to determine bivariate correlation analyses. All data are presented as mean ± SEM, and differences were determined statistically significant at a *p* value < 0.05.

## 3. Results

### 3.1. Subject Characteristics

Cognition groups were weighted equally with T2DM participants. There were no significant differences between cognition groups with respect to fasting glucose, fasting insulin, or HOMA-IR (Table 1). There was also no difference in age or lean body mass between groups; however, the AD group did have a significantly lower BMI (*p* = 0.03) and fat mass (*p* < 0.01) compared to the NC group (Table 1).

### 3.2. The Effect of Alzheimer's Disease on sRAGE Isoforms

**Figure 1:**
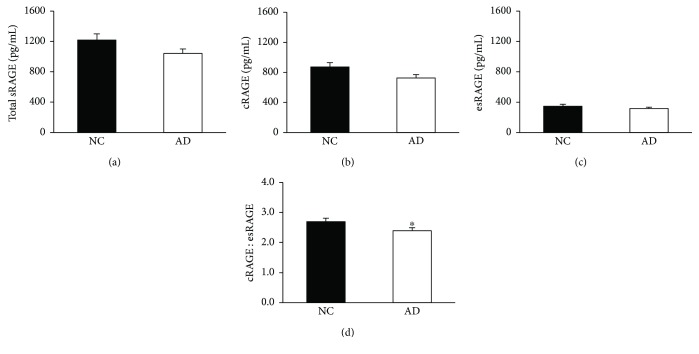
Attenuated cRAGE : esRAGE ratio with Alzheimer's disease compared to individuals with normal cognition. (a) Total sRAGE and (c) esRAGE were measured using commercially available ELISAs. (b) cRAGE was calculated by subtracting esRAGE from total sRAGE and the (d) cRAGE : esRAGE ratio was calculated by division. Data are presented as mean ± SEM. Sample sizes for the stratifications are as follows: NC (*n* = 68) and AD (*n* = 67). ^∗^*p* < 0.05 versus NC.

There were no significant differences in total serum sRAGE between the AD and NC groups; however, individuals with AD had a significantly reduced cRAGE : esRAGE ratio (*p* = 0.01) compared to those with NC (Figures 1(a) and 1(d)). Although the cRAGE and esRAGE isoforms alone were not significantly different between groups (*p* = 0.07 and 0.79, resp.), trending suggests that the 14.4% attenuation in the ratio is likely due to a preferential loss in cRAGE rather than esRAGE (Figures 1(b) and 1(c)).

### 3.3. The Effect of AD, Age, and APOE4 Genotype on sRAGE Isoforms

Given the altered sRAGE profile with AD presented in Figure 1, we aimed to identify any effects that age or genotype may have on serum sRAGE isoforms. First, stratification by decade of age (60–69, 70–79, 80–89 y) revealed a main effect of AD on cRAGE (*p* = 0.03) but no significant differences between groups for total sRAGE, esRAGE, or the cRAGE : esRAGE ratio. We next stratified by APOE4 genotype, the primary risk gene in AD [34], and found significant main effects of AD on total sRAGE, cRAGE, and the cRAGE : esRAGE ratio (*p* = 0.05, 0.02, and 0.01). There were no main effects of age or genotype nor any interactions between the above factors and AD status on any of the sRAGE variables. These findings suggest that age and APOE4 genotype may be important factors in the diminished cRAGE with AD.

### 3.4. The Effect of AD and Metabolic Dysfunction on sRAGE Isoforms

To better characterize the effects of metabolic factors on sRAGE pathophysiology in AD, all participants were stratified by the following factors: T2DM diagnosis, FGS as determined by ADA criteria, obesity by BMI according to the WHO, median insulin, median amylin, median HOMA-IR, and median fat mass. The above factors were selected for investigation due to their independent importance in disease pathophysiology and sRAGE biology. For example, we have previously found that glucose tolerance and obesity have a strong influence on sRAGE [29] and that adiposity (fat mass) is associated with attenuated sRAGE pools [35]. Further, this project aimed to characterize sRAGE profiles by cognitive status and commonly used markers of metabolic health to serve as a foundation for future work.

Total serum sRAGE and esRAGE were not significantly different between individuals with or without T2DM comorbid with AD. However, there was a significant main effect of AD on serum cRAGE and the cRAGE : esRAGE ratio (*p* = 0.03, 0.03), showing decreased levels of these sRAGE isoforms with AD (data not shown). Similarly, when stratified by FGS, there was a main effect of AD, which highlighted lower total sRAGE and cRAGE compared to NC counterparts (*p* = 0.04, 0.02; data not shown).

**Figure 2:**
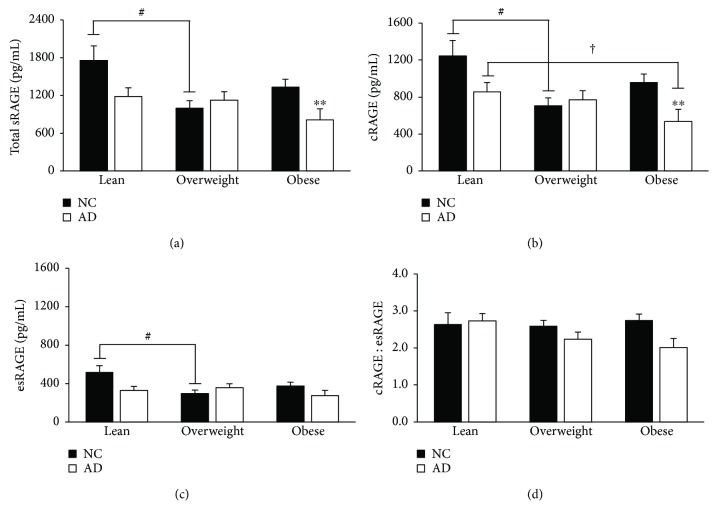
Diminished sRAGE pools are characteristic of individuals with concurrent AD and obesity. Participants were grouped by obesity status (lean: <25 kg/m^2^, overweight: 25–29 kg/m^2^, obese: ≥30 kg/m^2^), and sRAGE isoforms were measured in pg/mL as mentioned previously. Data are presented as mean ± SEM. Sample sizes for the stratifications are as follows: lean, NC (*n* = 7), lean, AD (*n* = 19), overweight, NC (*n* = 27), overweight, AD (*n* = 20), obese, NC (*n* = 24), and obese, AD (*n* = 12). ^#^*p* value < 0.05 across metabolic factor within NC group; ^†^*p* value < 0.05 across metabolic factor within AD group; ^∗∗^*p* value < 0.01 across groups within metabolic factor.

**Figure 3:**
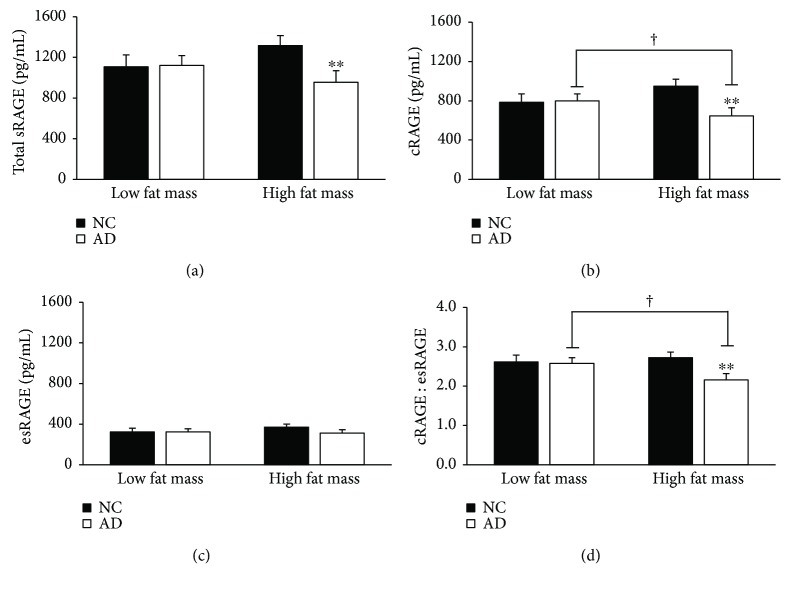
AD with high fat mass revealed marked reductions in total sRAGE and cRAGE and cRAGE : esRAGE compared to NC with high fat mass. Participants were stratified by median fat mass (29 kg) measured via DEXA. Data are presented as mean ± SEM. Sample sizes for the stratifications are as follows: low fat mass, NC (*n* = 26), low fat mass, AD (*n* = 37), high fat mass, NC (*n* = 37), and high fat mass, AD (*n* = 28). ^†^*p* value < 0.05 across metabolic factor within AD group; ^∗∗^*p* value < 0.01 across groups within metabolic factor.

Obesity stratification by BMI revealed main effects of AD on total sRAGE, cRAGE, and cRAGE : esRAGE ratio (*p* = 0.01, <0.01, and 0.02), main effects of obesity status on total sRAGE and cRAGE (*p* = 0.02, 0.01), and interaction effects on total sRAGE, esRAGE, and cRAGE (*p* = 0.02, 0.02, and 0.02). Post hoc analysis showed that within the NC group, those who are overweight have significantly lower total sRAGE, cRAGE, and esRAGE than their lean counterparts (*p* = 0.01, 0.02, and 0.02; Figure 2). Similarly, within the AD group, obese individuals have significantly reduced cRAGE compared to lean individuals (*p* = 0.03; Figure 2(b)). Those with comorbid obesity and AD have significantly lower total sRAGE and cRAGE compared to obese NC individuals (*p* = 0.01, <0.01; Figure 2). When stratified by median fat mass (28.95 kg), the AD group had lower serum cRAGE and cRAGE : esRAGE (*p* = 0.04, 0.02; Figure 3). Interaction effects between AD status and fat mass highlighted significant attenuation of total sRAGE, cRAGE, and the cRAGE : esRAGE ratio with AD compared to NC in the high fat mass group (*p* = 0.01, <0.01, and <0.01; Figure 3). As shown in Figure 3, individuals in the AD group with high fat mass had 4.1% less cRAGE and a 21.2% reduction in the cRAGE : esRAGE ratio compared to their counterparts with low fat mass (*p* = 0.3, 0.02).

**Figure 4:**
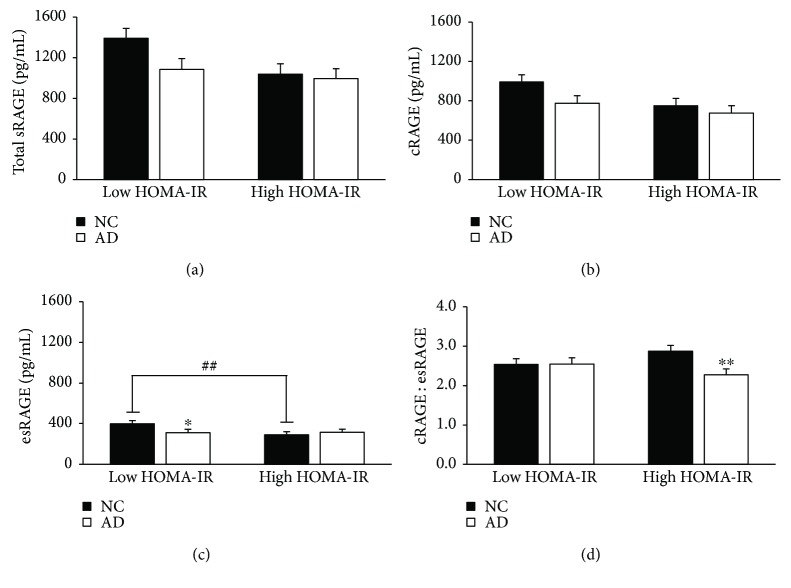
Decreased esRAGE and cRAGE : esRAGE ratio with high HOMA-IR or AD. HOMA-IR was calculated from fasting measures of glucose and insulin, and participants were stratified by the median HOMA-IR (1.7 AU). Data are presented as mean ± SEM. Sample sizes for the stratifications are as follows: low HOMA-IR, NC (*n* = 35), low HOMA-IR, AD (*n* = 30), high HOMA-IR, NC (*n* = 33), and high HOMA-IR, AD (*n* = 35). ^##^*p* value < 0.01 across metabolic factor within NC group; ^∗^*p* value < 0.05 across groups within metabolic factor; ^∗∗^*p* value < 0.01 across groups within metabolic factor.

HOMA-IR was calculated as an indicator of insulin resistance, and participants were stratified by the calculated median (1.66 AU). This data showed a significant difference between the AD and NC groups in serum cRAGE and the cRAGE : esRAGE ratio (*p* = 0.04, 0.02; Figure 4). Additionally, within the high HOMA-IR group, individuals with concurrent AD had a 25.7% lower cRAGE : esRAGE ratio (*p* < 0.01; Figure 4). Fasting HOMA-IR was also negatively correlated to log-transformed serum cRAGE (*r* = −0.19, *p* = 0.03).

**Figure 5:**
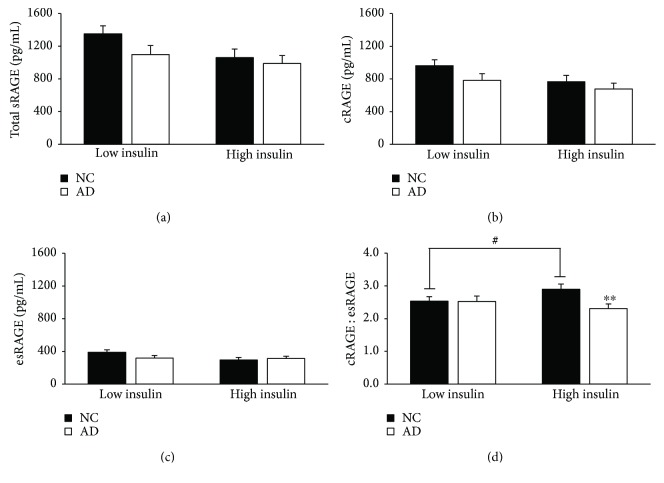
Individuals with high insulin concurrent with AD display attenuated cRAGE : esRAGE. Serum insulin was measured in mU/L following an overnight fast, and participants were stratified by the calculated median (6.83 mU/L). Data are presented as mean ± SEM. Sample sizes for the stratifications are as follows: low insulin, NC (*n* = 37), low insulin, AD (*n* = 31), high insulin, NC (*n* = 28), and high insulin, AD (*n* = 37). ^#^*p* value < 0.05 across metabolic factor within NC group; ^∗∗^*p* value < 0.01 across groups within metabolic factor.

**Figure 6:**
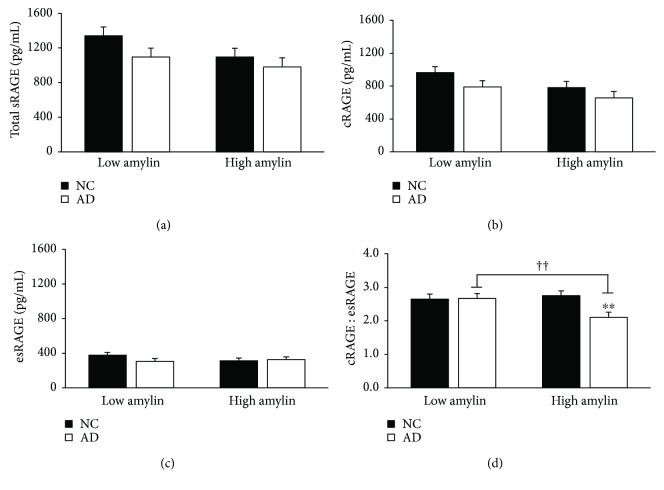
Aberrant cRAGE : esRAGE in individuals with high amylin and comorbid AD. Serum amylin was measured in pM following an overnight fast, and participants were stratified by the calculated median (6.8 pM). Data are presented as mean ± SEM. Sample sizes for the stratifications are as follows: low HOMA-IR, NC (*n* = 34), low HOMA-IR, AD (*n* = 34), high HOMA-IR, NC (*n* = 34), and high HOMA-IR, AD (*n* = 32). ^††^*p* value < 0.01 across metabolic factor within AD group; ^∗∗^*p* value < 0.01 across groups within metabolic factor.

Insulin and amylin were found to have a similar influence on serum sRAGE profiles in AD, but amylin was of special interest due to its ability to colocalize with A*β* plaques in AD [36]. Outlined in Figures 5 and 6, when stratified by median insulin (6.83 mU/L) and median amylin (6.75 pM), individuals with AD presented with lower cRAGE (*p* = 0.04, 0.03) and cRAGE : esRAGE (*p* = 0.01, 0.01). In addition, there was an interaction between AD and both insulin group and amylin group (*p* = 0.02, <0.01; Figures 5 and 6). Within both the high insulin and high amylin groups, those with concurrent AD displayed aberrant serum cRAGE : esRAGE ratios compared to NC counterparts, with a 25.6% and 30.1% attenuation, respectively (*p* < 0.01, <0.01). Related, fasting amylin was negatively correlated to log-transformed values of both the esRAGE (*r* = −0.31, *p* < 0.01) and the cRAGE isoforms (*r* = −0.29, *p* < 0.01). Fasting insulin was also negatively correlated to log-transformed serum cRAGE (*r* = −0.19, *p* = 0.03).

To understand any influence that the well-established factors of age and sex may have on AD-related outcomes, we ran ANCOVAs for these factors. All of the significant interactions we found between the experimental metabolic factors of interest and AD status on sRAGE profiles were maintained with covariance for age and sex (data not shown).

### 3.5. The Effect of AD on the sRAGE Ligand S100B

There were no significant differences in serum S100B between the AD and NC groups (Table 1). Ratios between serum S100B and serum total sRAGE, esRAGE, and cRAGE were generated to investigate RAGE ligand and sRAGE dynamics. Analyses showed no significant differences across AD diagnosis (data not shown). Additionally, there were no significant correlations among S100B, total sRAGE, or any of the sRAGE isoforms. We further investigated serum S100B against determinants of metabolic health (fat mass, BMI, insulin, glucose, and HOMA-IR) and found no significant relationships.

## 4. Discussion

To our knowledge, this is the first report to characterize total sRAGE and all sRAGE isoforms in individuals with and without Alzheimer's disease and comorbid T2DM. Our data show that individuals with metabolic derangement (elevated insulin, amylin, HOMA-IR, BMI, or fat mass) concurrent with AD present with aberrant sRAGE profiles. These findings are in line with other published work that shows decreased plasma sRAGE in individuals with cognitive impairment, obesity, and IR [26, 30, 37] and further supports the protective role of sRAGE in inflammatory diseases.

Novel findings of importance were the alterations in the sRAGE profile when individuals were stratified by AD diagnosis and median amylin. Fasting amylin was also negatively correlated to both esRAGE and cRAGE. Amylin readily crosses the blood-brain barrier and is known to play an important role in mediating brain function [38]. Further, amylin and A*β* share many chemical features, as both molecules bind to the same amylin-3 receptor in the brain [39]. Under pathological conditions, amylin can form insoluble amylin amyloid plaques, which have been found to colocalize with A*β* plaques in AD [36]. Abnormal amylin is central to T2DM progression as it is seen in both early stages (elevated amylin) and late stages (suppressed amylin) of the disease [40]. This, combined with the known relationship between RAGE and neurodegenerative disorders, highlights the link between abnormal amylin, depressed sRAGE, and Alzheimer's disease seen in our results.

We also showed that when our analyses were covaried for both age and sex, all interaction effects between metabolic dysfunction and AD status remained significant. Similarly, when individuals were stratified by decade of age or APOE4 genotype, there were neither any main effects of these variables nor an interaction with AD status. Although APOE4 genotype and increasing age are well-established risk factors for late-onset AD, previous studies have found that total plasma sRAGE levels are able to predict cognitive decline seen in late-onset AD [30]. Together with our data, these findings suggest that widespread metabolic dysfunction, rather than more traditional risk factors, may be more important drivers of AD in some individuals.

However, when we examined sRAGE in individuals with AD, T2DM status did not impact total sRAGE or any specific isoforms. This differs from previous work out of our lab and others that suggest diminished sRAGE pools in individuals with T2DM compared to healthy controls [29, 41]. There is also conflicting evidence demonstrating that increased total sRAGE is associated with diabetes [42]. One possible explanation for this difference may be that in the present, all study participants remained on their T2DM medication, whereas Basta et al. only included T2DM subjects without medication [41]. Another possible explanation is that many of these volunteers may be newly diagnosed with T2DM or have adequate glycemic control as the median HOMA-IR of our T2DM participants was 2.45, much lower than previous established cutoffs [43]. Lastly, it may be possible that AD pathology may present similarly to T2DM and thus may mask any unique effects of T2DM on sRAGE isoforms.

S100B is thought to mediate neuroinflammation and contribute to both AD pathogenesis and systemic inflammation seen in metabolic disease. Importantly, S100B is overexpressed in the brain of AD patients [44, 45] and elevated in serum of persons with obesity [46]. However, reports of S100B in cerebral spinal fluid (CSF) and serum of AD patients are conflicting. Peskind et al. reported elevated CSF S100B in individuals with mild to moderate AD compared to controls [47], while Nooijen et al. found no difference in CSF S100B with dementia [48]. Chaves et al. reported lower serum S100B in AD compared to controls [49], which differs with our cross-sectional analysis, as we did not see any differences in serum S100B across AD status. A possible explanation of our findings is that elevated RAGE in the AD brain sequesters and traps S100B proteins in brain tissue, preventing it from appearing in the serum despite the compromised blood-brain barrier in AD. Similar RAGE trapping mechanisms have been previously reported in adipose tissue for advanced glycated end products [50]. Further, adipose tissue serves as a site of S100B protein concentration comparable to neuronal tissue [51, 52]. S100B secretion from adipocytes is related to lipolysis [53], inhibited by insulin [54], and thus is subject to dysregulation by diabetes and other metabolic diseases. This may further explain our findings as we balanced the number of T2DM participants across cognitive groups. These data further illuminate the need to consider metabolic covariates when assessing neurocognitive disorders. Postmortem analysis of brain sections combined with CSF and serum analyses may help to clarify the role and origin of S100B in the pathogenesis of AD.

In conclusion, the following metabolic factors have been shown to influence total sRAGE and individual sRAGE isoforms in Alzheimer's disease: elevated insulin, amylin, insulin resistance as described by HOMA-IR, obesity by BMI, and high fat mass. It is possible that the attenuated plasma sRAGE seen in individuals with metabolic dysfunction may be contributing to AD due to a reduced capacity to scavenge RAGE ligands and attenuate RAGE signaling. Activation of RAGE produces oxidative stress and inflammation in the brain, neuronal degeneration, and A*β* accumulation, all inducers of cognitive impairment and AD [10, 20]. These novel characterizations of sRAGE in individuals with concurrent metabolic derangement and AD contribute evidence to the link between impaired metabolism and cognitive decline.

## Figures and Tables

**Table 1:** Subject characteristics.

Variable (units)	NC (*n* = 68)	AD (*n* = 67)
Sex (number, % males)	26, 38%	36, 54%
Age (y)	73 ± 1	75 ± 1
BMI (kg/m^2^)	29.5 ± 0.5	27.3 ± 0.8^∗^
Lean body mass (kg)	46 ± 1	46 ± 1
Fat mass (kg)	32 ± 1	27 ± 1^∗^
Type 2 diabetes (number, %)	22, 32%	19, 28%
HOMA-IR (AU)	2.4 ± 0.3	2.2 ± 0.2
Fasting glucose (mg/dL)	104 ± 3	101 ± 2
Fasting glucose (mmol/L)	5.8 ± 0.2	5.6 ± 0.1
Fasting insulin (mU/L)	9.1 ± 1.0	8.8 ± 0.8
Fasting amylin (pM)	11.2 ± 1.7	17.9 ± 5.3
S100B (pg/mL)	13.0 ± 1.7	14.4 ± 2.8

Data are presented as mean ± SEM. BMI: body mass index; HOMA-IR: homeostatic model assessment of insulin resistance. ^∗^*p* < 0.05.
